# A Comparative Study between the Three Waves of the Pandemic on the Prevalence of Oropharyngeal Dysphagia and Malnutrition among Hospitalized Patients with COVID-19

**DOI:** 10.3390/nu14183826

**Published:** 2022-09-16

**Authors:** Paula Viñas, Alberto Martín-Martínez, Claudia Alarcón, Stephanie A. Riera, Jaume Miró, Cristina Amadó, Pere Clavé, Omar Ortega

**Affiliations:** 1 Gastrointestinal Physiology Laboratory, Hospital de Mataró, Department of Medicine, Universitat Autònoma de Barcelona, 08304 Barcelona, Spain; 2 Centro de Investigación Biomédica en Red de Enfermedades Hepáticas y Digestivas (CIBERehd), 28029 Madrid, Spain

**Keywords:** swallowing disorders, oropharyngeal dysphagia, COVID-19, malnutrition, nutritional risk, fluid thickening

## Abstract

Background: The phenotype of patients affected by COVID-19 disease changed between the waves of the pandemic. We assessed the prevalence of oropharyngeal dysphagia (OD), malnutrition (MN), and mortality between the first three waves of COVID-19 patients in a general hospital. Methods: a prospective observational study between April 2020–May 2021. Clinical assessment for OD was made with the volume-viscosity swallowing test; nutritional assessment was performed consistent with GLIM criteria. A multimodal intervention was implemented in the second and third wave, including (a) texturized diets—fork mashable (1900 kcal + 90 g protein) or pureed (1700 kcal + 75 g protein), (b) oral nutritional supplements (500–600 kcal + 25–30 g protein), and (c) fluid thickening (250 mPa·s or 800 mPa·s). Results: We included 205 patients (69.3 ± 17.6 years) in the 1st, 200 (66.4 ± 17.5 years) in the 2nd, and 200 (72.0 ± 16.3 years;) in the 3rd wave (*p* = 0.004). On admission, prevalence of OD was 51.7%, 31.3% and 35.1%, and MN, 45.9%, 36.8% and 34.7%, respectively; mortality was 10.7%, 13.6% and 19.1%. OD was independently associated with age, delirium, and MN; MN, with age, OD, diarrhea and ICU admission; mortality, with age, OD and MN. (4) Conclusions: Prevalence of OD, MN and mortality was very high among COVID-19 patients. OD was independently associated with MN and mortality. An early and proactive multimodal nutritional intervention improved patients’ nutritional status.

## 1. Introduction

The World Health Organization declared a global public emergency due to the Severe Acute Respiratory Syndrome Coronavirus (SARS-CoV-2) epidemic and the disease it causes, COVID-19 [1]. It was declared a pandemic in spring 2020, and caused high rates of both severely ill patients and mortality [2,3]. Fever, cough, sore throat, breathing difficulties and fatigue were the most common symptoms of the disease [2,3]. Its most common risk factors were age, the environment, unhealthy diets and lifestyles and the presence of previous chronic diseases such as diabetes, hypertension, obesity and immune system disorders. In sum, 80% of patients presented mild symptoms, 15% became grave, and finally 5% became critical with respiratory distress syndrome and multiorgan failure [4,5].

SARS-CoV-2 infection leads to a cytokine storm from different mechanisms, such as the activation of interleukins and macrophages. It generates an increased energy expenditure and the disruption of other mechanisms, adversely affecting the immune system and tissue repair [4,6]. Inadequate nutrition is common in these patients, particularly in the critically ill whose nutritional requirements are high, a condition known as hypercatabolism. Disease-related malnutrition (MN) is related to increased mortality, longer hospital stay, especially those in intensive care units (ICU), and increased morbidity on discharge [7].

Oropharyngeal dysphagia (OD) is a common complication in COVID-19 patients [8,9]. Some authors have suggested that polyneuropathy, myopathy and other neurological impairments that are frequent developments after COVID-19 [6,10], together with the invasion of peripheral nerves by SARS-CoV-2 causing ageusia and anosmia and pharyngeal sensory dysfunction, could be associated with the development of OD [6,10,11,12]. In addition, another important mechanism could be the loss of swallowing muscle mass and strength known as sarcopenic dysphagia [13,14], which can be evaluated with several tools, including muscle mass, muscle strength and physical performance [15]. Sarcopenic dysphagia has been described in non-intubated elderly patients with severe COVID-19 infection [16]. In a previous study on COVID-19 patients admitted to a general hospital during the first wave, we found a high prevalence of OD (51.7%) and MN (45.5%) during admission, with a mean weight loss (WL) of 10.1 ± 5.0 kg during hospitalization. In our study, OD was independently associated with comorbidities, neurological symptoms, and low functionality; in addition, it was associated with increased 6-month mortality (28.4% OD vs. 7.1% no-OD, *p* < 0.001). Currently, there are no studies comparing swallowing and its complications, including malnutrition and mortality, between the different waves of the pandemic. The aim of this study is to compare the prevalence of OD, MN and mortality rate between the three waves of the pandemic and the percentage of WL during hospitalization following the introduction of a multimodal intervention including fluid thickening, texture modified foods and nutritional support among patients with COVID-19 at Mataró Hospital, Catalonia, Spain.

## 2. Materials and Methods

### 2.1. Study Population

Patients with COVID-19 consecutively admitted to Mataró Hospital during the acute phase and to Sant Jaume i Santa Magdalena Hospital (Consorci Sanitari del Maresme [CSdM]) were the study population. All SARS-CoV-2-positive patients were prospectively assessed from April 2020 to May 2021, divided by the three waves of the pandemic: 1st wave, from 14 April 2020 to 30 July 2020; 2nd wave, from 3 August 2020 to 31 December 2020; and 3rd wave, from 4 January 2021 to 19 May 2021. COVID-19 disease was confirmed by reverse transcription polymerase chain reaction [RT-PCR] with GeneXpert Dx (Cepheid, Sunnyvale, CA, USA) and those patients who could be assessed for OD and nutritional status within the first 2 days of hospitalization were included. If they died in the ICU without prior assessment or if a swallowing and/or nutritional assessment was not possible, they were excluded.

### 2.2. Study Design

The present work is a prospective observational and comparative quasi-experimental study including all COVID-19 patients hospitalized at the CSdM more than 2 days. We collected demographic, clinical, swallowing and nutritional data from the participants on pre-admission, admission, during stay and on discharge. Following the previous management protocol designed by Clave et al. 2020 [17] and according to what had been done in the 1st wave [8], the information was collected telematically from COVID-19 wards (to minimize the risk of cross-infection) by telephone or videoconference directly with the patient, their family or their nurses/physicians, as well as from the patient’s medical records as previously reported [8]. A multidisciplinary team worked together to collect study variables for clinical (nurse/physician), swallowing (speech language pathologist [SLP] and nurses) and nutritional (dietitian and nutritionists) assessments.

The study protocol was conducted according to the principles and rules laid down in the Declaration of Helsinki and its subsequent amendments and it was approved by the Institutional Review Board of the Hospital de Mataró (CEIm 34/20). The informed consent form was granted an exemption by the Ethics Committee from the CSdM and followed the Guidance on the Management of Clinical Trials during the COVID-19 pandemic (European Commission, version 4; 4 February 2021). ClinicalTrials.gov Identifier: NCT04346212.

### 2.3. Clinical Assessment

Patients’ clinical information was collected on (a) admission (patient’s residence (from the community, nursing home or intermediate care hospital); pre-COVID-19 and admission functional status (Barthel Index) [18,19]; comorbidities assessed by Charlson index [20] and COVID-19 symptoms during the days prior to admission), (b) hospitalization (length of hospital stay; clinical and neurological symptoms; respiratory scales to measure insufficiencies and severity; use and concentration of oxygen therapy; admission and length of stay in the ICU and neuromyopathy development; pronation requirements; pharmacological treatment) and (c) discharge (patient’s destination; functional status; diagnostic codes on discharge such as pneumonia caused by SARS-CoV-2 infection, aspiration pneumonia [21,22], respiratory or other bacterial infections [23], OD, MN and mortality during hospitalization).

### 2.4. Swallowing Assessment

Telematic explorations were used to evaluate swallowing disorders and masticatory dysfunctions of patients admitted to COVID-19 wards. Questions focused on impaired chewing function with solid food, as well as a specific anamnesis of clinical signs and symptoms of OD. Specialized SLPs used the Eating Assessment Tool-10 (EAT-10) [24] to screen patients at risk for dysphagia (a score ≥ 2 indicates risk of swallowing disorders) [24,25] and, with the support of the nursing staff [17], a simplified volume-viscosity swallowing test (V-VST) [26] for clinical assessment of OD in COVID-19 patients using the three usual viscosities (liquid, 250 mPa·s and 800 mPa·s) but adapted to a single bolus volume of 10 mL at each viscosity [8]. Viscosities were prepared with the thickening product Nutilis Clear (Nutricia N.V., Zoetermeer, The Netherlands) in accordance with our previous studies [27].

### 2.5. Nutritional Assessment

Nutritional evaluation was performed in two steps according to the recommendation of the Global Leadership Initiative on Malnutrition (GLIM) [28], first, on admission, to identify the risk with the Nutritional Risk Screening 2002 (NRS-2002) score, which has recently been recommended as the best-validated option to identify the risk of MN for hospitalized adult COVID-19 patients [29]; and second, during hospitalization, with assessment for diagnosis and grading of the severity of malnutrition according to GLIM (it requires at least 1 phenotypic and 1 etiologic criterion) [30]. Anthropometric measurements such as weight (kg), percentage of WL, height (cm) and body mass index (BMI [kg/m^2^]) were also collected. Other information was also collected from pre-admission, during hospital stay and on discharge, such as diet intake and appetite, nutritional recommendations (prescription of oral nutritional supplements (ONS) and type of diet) and patient’s adherence to them. The use of non-oral nutrition was also collected. Blood analytical parameters were registered on admission and discharge when available (total lymphocytes, ferritin, *C*-reactive protein (CRP), albumin, cholesterol and total proteins). The Reference Laboratory of Catalonia reference intervals were used [31], the normal and standard values being: lymphocytes, 1 × 10^3^–3 × 10^3^/µL; ferritin, 30–400 ng/mL; CRP, <0.5 mg/dL; albumin, 3.5–5.2 g/dL; cholesterol, 120–200 mg/dL, and total proteins, 6–8.3 g/dL.

### 2.6. Nutritional Intervention

**Figure 1:**
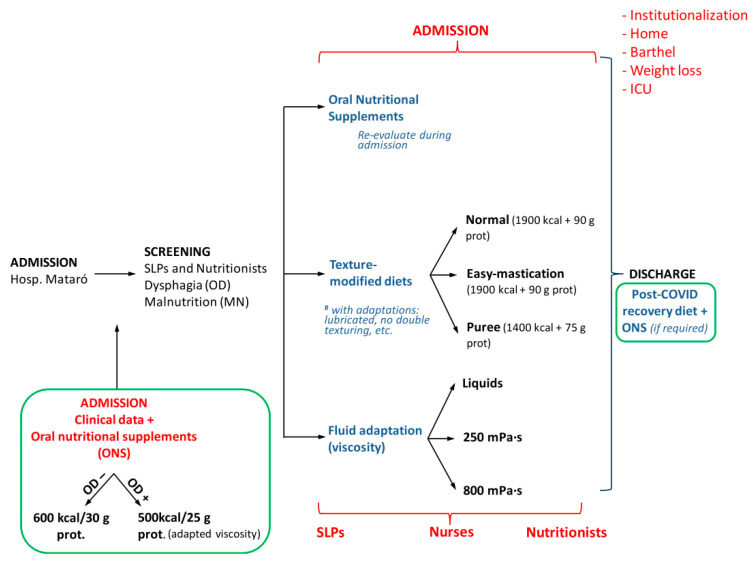
Management of patients with COVID-19 disease, oropharyngeal dysphagia and risk of malnutrition. In green, the new management directives introduced in the second and third waves.

Patients received a diet based on the triple adaptation of the Mediterranean diet [32], one that met nutritional needs, and that had been adapted in texture and fluids, according to the patients’ mastication and swallowing function and nutritional status [32]. The textural adaptation included: (a) three levels of food texture—normal texture, fork-mashable, or pureed diets [32]; and (b) three levels of shear viscosity for fluids—“liquid” < 50 mPa.s, “nectar” 250 mPa·s, and “honey” 800 mPa·s. The food was then prepared with 0, 2 and 5.5 g of Nutilis Clear (Nutricia N.V., Zoetermeer, The Netherlands), respectively, in 100 mL water [27]. During the first wave of the pandemic, patients with a normal nutritional status received the standard hospital diet (1750 kcal + 70–80 g proteins) and those who received a nutritional assessment and required some type of nutritional support received ONS following nutritional and rheological assessment (“assess and treat”) [8]. Not all were able to receive a nutritional evaluation, and therefore did not receive ONS. During the 2nd and 3rd waves, a systematic nutritional intervention (“screen and treat”) was implemented in all hospitalized patients, on admission, consisting of: (a) hypercaloric-hyperproteic diet (normal texture diet and fork mashable diet [1900 kcal + 90 g protein] or pureed [1700 kcal + 75 g protein]); (b) ONS (2 units) of the type used at our hospital, with the following health registration number: 26.10319/B-08599, one with low viscosity (each bottle 200 mL and 300 kcal + 15 g protein) and the other with high viscosity (each one 125 g and 250 kcal + 12.5 g protein) according the dysphagia status, and (c) recommendations for a hypercaloric-hyperproteic diet on discharge (Supplementary File S1). The low viscosity ONS is a high-calorie and high-protein supplement with fiber and it is available for diabetic patients or those with alterations in glycaemia, a population prevalent among COVID-19 patients due to corticotherapy. Therefore, an extra 500–600 kcal and 25–30 g of protein per day was given at admission to patients in the 2nd and 3rd wave following this new “screen and treat” strategy. Nutritional status was re-evaluated during hospitalization, and nutritional management was adapted according to these evaluations. Texture-modified diets provided during admission were manufactured by Arcasa SL (Esplugues de llobregat, Barcelona, Spain) at two levels of textural adaptation (fork mashable vs. pureed). The algorithm used is detailed in Figure 1.

### 2.7. Data Management and Statistical Analysis

Prevalence of OD, risk and prevalence of MN, total WL during hospital admission, and intrahospital mortality were the main outcome study variables. We also aimed to assess whether patients with OD and those with OD and MN had a worse prognosis compared with those without these conditions. Two analyses were carried out: an initial descriptive analysis of all 605 patients included in the three waves of the pandemic, and a subsequent comparative analysis between the three waves.

Qualitative data were presented as relative and absolute frequencies analyzed using Fisher’s exact test or the Chi-square test. Continuous data were presented as mean standard deviation (SD) and compared using the *t*-test (between-group comparisons) or paired *t*-test (within-group comparisons). For variables that did not follow a normal distribution, we used the non-parametric Mann-Whitney U test (between-group comparisons), the Wilcoxon test (within-group comparisons) or the Kruskal-Wallis test for multiple comparisons with Dunn’s multiple comparisons test. To assess normality, we used the D’Agostino and Pearson omnibus normality test.

For bivariate analysis, the Chi-square test was used to assess the relationships between the different categorical factors with OD and MN (on discharge) and mortality (during hospital stay). For continuous factors, Student’s *t*-test (normal distribution) and the Mann-Whitney U-test (non-normal distribution) were used. Multivariate models were performed with significantly associated factors (*p* < 0.05) and those clinically relevant to the different outcomes. The Stepwise method was used to assess independent factors. Functionality and weight change during the study period were calculated and plotted with data from surviving patients.

Results were interpreted according to the *p*-value obtained, the magnitude of the observed effect and its clinical and biological plausibility. Statistical significance was accepted for *p*-values < 0.05. Statistical analysis was performed with the specific language R (R Project for Statistical Computing; www.r-project.org; accessed on 15 September 2022).

## 3. Results

### 3.1. Demographics and Clinical Characteristics of the Study Population

#### 3.1.1. Total Participants

**Figure 2:**
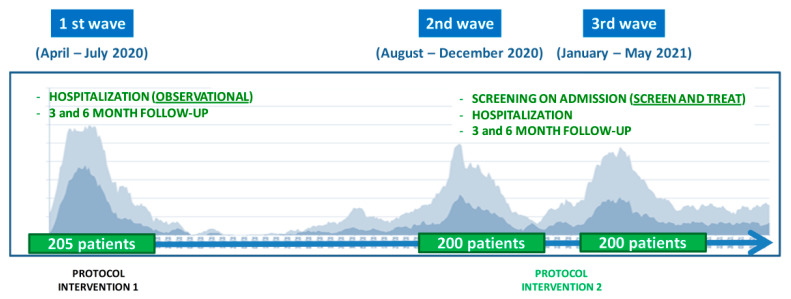
Curves of admission during the three waves of the pandemic at Consorci Sanitari del Maresme, Catalonia, Spain. A dark gray shadow depicts hospitalization, and a light gray shadow depicts visits to the emergency department.

We included 605 patients (Figure 2) with a positive SARS-CoV-2 RT-PCR hospitalized at some point at CSdM from April 2020 to September 2021, with a mean age of 69.2 ± 17.3 years, 49.9% female. Most of them came from the community (80.2%), the rest came from nursing homes (18%) and intermediate care centers (1.8%). Following discharge, they were referred to nursing homes (15.2%; *p* < 0.001) and to intermediate care centers (8.8%; *p* < 0.001). Patients presented a moderate dependence at pre-admission (BI: 84.0 ± 26.6) which worsened significantly upon hospitalization and discharge (admission BI: 75.9 ± 33.2 and discharge BI: 82.2 ± 29.7; *p* < 0.001). The main symptoms suffered by COVID-19 patients in the days prior to admission were fever (66.9%), cough (50.4%) and dyspnea (44.3%), followed by diarrhea (20.2%), vomiting (7.9%), headache (19%), ageusia (15.5%) and anosmia (12.4%). The main neurological symptoms during admission were confusion (27.8%), headache (23.5%), delirium (8.8%), ageusia (3.1%), anosmia (2.2%) and encephalitis (0.8%).

Patients were hospitalized during a mean period of 12.7 ± 10.9 days. Up to 62% suffered from interstitial pneumonia, followed by a suspected bacterial infection in 10.8% of cases. Maximum mean fraction of inspired oxygen (FIO2) during hospitalization was 48.3 ± 31.3, 28.2% of them requiring high-flow oxygen venturi masks and 24.1% high concentration oxygen masks. In most cases, pharmacological treatment included antibiotics (42.9%); 8.2% of patients were referred to the ICU for a mean stay of 15.5 ± 14.0 days; 6.5% required orotracheal intubation for 14.4 ± 14.3 days, and overall intra-hospital mortality was 14.4%.

#### 3.1.2. Three Waves Description

**Figure 3:**
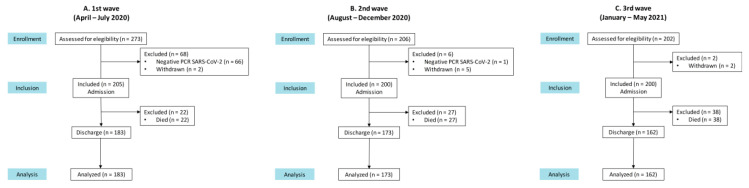
Consort study flow chart for patients included in the study in each of the first three waves of the pandemic. (**A**) first wave; (**B**) second wave; (**C**) third wave.

In the first wave, 205 patients were included, compared with the first 200 consecutively admitted patients in each one of the second and third waves (Figure 3).

Demographic data of all patients included in each wave is detailed in Table 1. We found statistical differences with regard to age, particularly when comparing the second and third waves (*p* = 0.004), with the oldest patients in the third wave. The majority of patients from the second and third waves came from the community, while the percentage of patients that came from nursing homes and social health centers was higher in the 1st wave (*p* < 0.001 s and third vs. first wave). Patients from three waves had impaired functional status, though slightly improved in the second wave (*p* = 0.012 between waves) and only 51.1% to 63.0% of patients from the three waves were independent for daily living activities. Serious neurological symptoms were more prevalent during the first wave of the pandemic; confusion affected 40.0%, 14.6%, and 28.5% of patients in the first, second and third waves, respectively (*p* < 0.001); headache in 28.3%, 24.6%, and 17.5%, *p* = 0.034, and delirium in 15.1%, 3.5%, and 7.5%, *p* < 0.001.

Clinical characteristics and the therapeutic approach of the three waves of COVID-19 patients is described in Table 2. The main differences between waves show a higher need for high-flux Venturi masks in the first and second waves (*p* < 0.001). The mean peak FIO2 administered in the second and third waves was significantly higher than in the first, although the ratio between oxygen arterial pressure (PaO2) and the FIO2 (PAFI) was greater in the first wave. The percentage of patients admitted to the ICU was higher in the first wave (*p* < 0.05) with a higher rate of orotracheal intubation (*p* = 0.009) than in the second wave, where an increase in the use of nasal intermittent positive pressure ventilation was observed (*p* < 0.05). Patients in the first wave had longer hospital stay (*p* < 0.001). Intra-hospital mortality was high among the three waves, especially in the third one (19.1%; *p* < 0.05 vs. 1st wave).

### 3.2. Swallowing and Masticatory Function

#### 3.2.1. Total Participants

Of 605 patients, only 10.6% were previously diagnosed with OD. On admission, 37.1% of patients referred to eating and/or drinking difficulties after the specific anamnesis and evaluation of OD. After the clinical assessment of OD with the V-VST, 39.6% were diagnosed with OD on admission, with 38.5% and 31.4% of patients presenting clinical signs of impaired efficacy and safety of swallow among the whole study population, respectively. Fluid adaptation was needed in 29% (35.8% at medium viscosity (250 mPa·s) and 3.8% at high viscosity (800 mPa·s) and diet, in 41.1% (23.4% fork-mashable and 27.3% pureed diet) of patients, respectively. On discharge, the prevalence of OD was slightly reduced at 35.7% (n = 210), as well as the need for fluid adaptation (14.8%) and texture modified diets (16.4%).

#### 3.2.2. Three Waves Description

The swallowing function of patients from the three waves of COVID-19 is described in Table 3. Patients in the first wave reported significantly greater difficulties in eating and/or swallowing on admission (EAT-10 and OD anamnesis). After evaluation using the V-VST, 51.7% of patients from the first wave had OD on admission, vs. 31.3% and 35.1% in the second and third waves, respectively (*p* < 0.001). On discharge, OD prevalence was reduced in all waves but remained higher in patients from the first (*p* = 0.006) (Table 3). Consequently, more patients in the first wave needed adaptation of fluids to medium viscosity on admission (*p* < 0.001) and to medium and high viscosity on discharge (*p* = 0.012) to avoid aspirations. No significant differences were observed with respect to prescription of texture modified diets either on admission or discharge between the three waves.

### 3.3. Nutritional Status

#### 3.3.1. Total Participants

Almost all COVID-19 patients included in this study (98.9%) were at risk of MN on admission according to NRS2002 (score ≥ 3). On discharge, 39.1% were malnourished according to GLIM criteria. Most of the patients on admission were overweight (mean BMI = 28.7 ± 5.7 kg/m^2^), but most of them reported having suffered WL before (63.0%) and during (62.0%) hospitalization, more specifically 2.1 ± 2.8 kg and 2.5 ± 4.5 kg, respectively. Total mean WL prior and during admission was 4.7 ± 5.2 kg. Only 26.6% (n = 114) of hospitalized COVID-19 patients conserved their appetite and ate a complete diet during the days prior to admission, which improved to 73.6% (n = 324) on discharge. ONS were prescribed to 74.0% with full adherence in only half of them (47.4%); 9.4% needed nasogastric tube feeding. Other prevalent symptoms during hospital stay that contributed to WL were: anorexia (23.8%, n = 107), vomiting and nausea (8.9%, n = 53), diarrhea (30.1%, n = 127) and incomplete diet intake (40.5%, n = 184).

#### 3.3.2. Three Waves Description

Almost all patients from the three waves presented high nutritional risk on admission, as measured with the NRS 2002. However, during hospitalization, the prevalence of MN was higher in patients from the first wave (*p* < 0.05 vs. third wave). Moreover those from the first wave had higher prevalence of clinical symptoms contributing to impaired nutritional status such as vomiting or nausea and diarrhea. In contrast, ONS prescription was significantly lower in the first wave (<0.001) and nasogastric tube placement higher (*p* < 0.001) (Table 4).

WL was significantly higher in the first wave with a higher percentage of patients losing more than 10 kg during hospitalization (19.2% first wave vs. 1.5% and 1.7% in the second and third waves, respectively; *p* < 0.0001) and a higher total WL from pre-admission to discharge (6.5 ± 5.8 kg 1st wave vs. 3.3 ± 4.2 kg and 4.1 ± 4.3 kg in the second and third waves respectively; *p* < 0.0001) (Table 5).

### 3.4. Biochemical Analysis

#### 3.4.1. Total Participants

On admission we observed an increase in acute phase reactants and inflammation such as ferritin and *C*-reactive protein, as well as a decrease in albumin to below reference values. However, the other analytical parameters such as cholesterol, total proteins and lymphocytes remained within reference values. We observed significant changes from admission to discharge in albumin (3.4 ± 0.4 mg/dL admission vs. 3.2 ± 0.6 mg/dL discharge; *p* < 0.001), total proteins (6.5 ± 0.6 g/dL admission vs. 6.2 ± 0.8 g/dL discharge; *p* < 0.001), lymphocytes (1.2 ± 0.9 × 10^3^/µL admission vs. 1.7 ± 1.6 × 10^3^/µL discharge; *p* < 0.001), ferritin (863.7 ± 1045.3 ng/mL admission vs. 1010.6 ± 3003.6 ng/mL discharge; *p* < 0.001) and CRP levels (8.5 ± 8.0 mg/dL admission vs. 3.3 ± 5.5 mg/dL discharge; *p* < 0.001). No significant changes in cholesterol were observed (135.8 ± 39.3 mg/dL admission vs. 167.3 ± 53.2 mg/dL discharge).

#### 3.4.2. Three Waves Description

Analytical parameters on admission and discharge in all the waves of the pandemic are detailed in Table 6. We observed significant differences in total proteins and lymphocytes on admission between the three waves. No significant differences were observed on admission with regard to cholesterol values; however, when patients were discharged, the ones in the second and third waves presented significantly lower values compared with the first wave (*p* < 0.05). Inflammatory status was higher in all the samples (according to CRP levels); however, on discharge, patients in the third wave presented significantly higher CRP compared with the first and second wave (*p* < 0.05). We didn’t observe any significant differences in albumin and ferritin values.

### 3.5. Risk Factors Associated with OD, MN and Mortality

#### 3.5.1. Oropharyngeal Dysphagia

Patients with OD were significantly older (OD: 81.7 ± 11.5 years vs. no-OD: 62.1 ± 15.7 years; *p* < 0.001), and had significantly worse functional status (BI), both prior to admission (63.4 ± 30.2 OD vs. 96.4 ± 12.0 no-OD; *p* < 0.001) and on discharge (51.9 ± 34.1 OD vs. 93.0 ± 18.7 no-OD; *p* < 0.001). Only 56.2% of patients with OD were hospitalized from the community, compared to 93.9% in non-OD patients (*p* < 0.001). The rest of patients with OD came from nursing homes (41.0%) and intermediate care centers (2.9%) (*p* < 0.001). Similarly, destination to the community on discharge was also significantly higher in patients without OD (OD: 43.4% vs. non-OD: 89.8%; *p* < 0.001). Up to 95% of patients with OD were at nutritional risk (NRS-2002 ≥ 3) compared to 73.3% in patients without (*p* < 0.001). In addition, patients with OD presented significantly higher prevalence of MN according to GLIM compared to patients without (OD: 54.0% MN vs. no-OD: 30.5% MN; *p* < 0.001). WL in both groups was similar, with no significant differences observed.

#### 3.5.2. Malnutrition

Patients with MN were also significantly older compared with patients without (OD: 73.1 ± 15.5 years vs. no-MN: 65.9 ± 17.5 years; *p* < 0.001), and presented significantly worse functional status, both pre-admission and on discharge (BI pre-admission: 81.6 ± 27.8 MN vs. 87.1 ± 24.4 no-MN; *p* = 0.008 and BI discharge: 78.9 ± 29.9 MN vs. 84.3 ± 29.3 no-MN; *p* < 0.001). No significant differences in MN prevalence were observed with regard to patients’ residence. OD was present in 48.2% of patients with MN, compared with 25.8% in patients without MN (*p* < 0.001), and consequently, required significantly more thickened fluids and TMD. Prescription of ONS was higher in MN patients (81.4% MN vs. 66.7% noMN; *p* < 0.001), as, per protocol especially in the second to the third wave, patients were treated with ONS to prevent further nutritional impairment. On discharge, only 59.1% of patients with MN ate a hundred percent of the diet, compared with 81.6% in patients without (*p* < 0.001). No significant differences were observed in any analytical parameters on admission except for CRP, where a significantly higher inflammatory status was observed in patients with MN (10.28 ± 9.20 mg/dL vs. 7.5 ± 7.1 mg/dL no MN; *p* < 0.001).

#### 3.5.3. Mortality

Mortality in the three waves was 10.7%, 13.5% and 20.8%, respectively (*p* < 0.05). COVID-19 patients who died during hospitalization were significantly older (82.62 ± 10.87 vs. 67.04 ± 17.11 years, respectively; *p* < 0.001), had worse functional capacity (59.89 ± 34.92 vs. 88.01 ± 22.54; *p* < 0.001), and more came from a nursing home (36.8% vs. 14.9%; *p* < 0.001) than those who survived. Patients who died had more impairments in swallow function requiring more adaptation of fluids with thickeners (64.3% vs. 23.2%; *p* < 0.001) and showed higher prevalence of previous OD (25.3% vs. 12.6%; *p* = 0.004) than those who survived. Regarding nutritional status, patients who died showed higher risk of MN on admission (96% vs. 78.6%; *p* < 0.001), lower mean weight pre-admission (68.9 ± 12.1 vs. 80.1 ± 18.2; *p* < 0.001) and higher needs for texture-modified diets (73.5% vs. 41.5%; *p* < 0.001). Blood analysis during hospital stay showed that those patients who died had lower levels of albumin (3.3 ± 0.5 vs. 3.4 ± 0.4 (g/dL); *p* = 0.017), total proteins (6.3 ± 0.7 vs. 6.6 ± 0.6 (g/dL); *p* = 0.003) and lymphocytes (0.9 ± 0.5 vs. 1.2 ± 0.9 (×10^3^/µL); *p* = 0.002) than those who survived. Finally, patients who died during hospital stay had a higher prevalence of OD (74.7% vs. 29.7% *p* < 0.001) and MN (76.9% vs. 33.3%; *p* < 0.001).

### 3.6. Multivariate Analysis: Independent Risk Factors Associated with OD, MN and Mortality

A multivariate logistic regression analysis showed that: (a) age, the presence of delirium, nutritional risk (NRS-2002 ≥ 3) and MN (GLIM criteria) were independently associated with OD (Table 7), all being risk factors for its development; (b) age, OD on admission, ICU admission, diarrhea and an incomplete diet on discharge were independently associated with MN during hospitalization (Table 7), and (c) age, OD on admission and MN (GLIM criteria) were independently associated with mortality during hospitalization (Table 7).

## 4. Discussion

The main aim of this study was to compare the prevalence of OD, MN and mortality between the three first waves of the pandemic in a general hospital and the impact of a systematic multimodal intervention, including fluid thickening, texture modified foods and nutritional support, on the clinical outcomes of these patients during hospitalization. We evaluated a total of 605 COVID-19 patients from the three first waves of the SARS-CoV-2 pandemic, a population constituted by older patients from the community with slightly impaired functionality and high prevalence of comorbidities. The main results found in the three waves showed that the prevalence of OD, MN and mortality was very high among hospitalized COVID-19 patients, that OD was independently associated with MN and mortality, and that an early, systematic and proactive multimodal nutritional intervention, “screen and treat,” improved patients’ nutritional status in the second and third wave.

The pathophysiology of OD in COVID-19 has been extensively studied and related to aging, neurological diseases, respiratory insufficiency, invasive respiratory support, sarcopenia and cachexia [33,34]. Sarcopenic dysphagia has been defined as a swallowing disorder due to sarcopenia involving the whole-body skeletal muscles and swallowing muscles [13]. It is characterized by decreased swallowing function [15], tongue strength and range of tongue motion, weakened pharyngeal muscle contraction and deteriorated endurance of swallowing muscles, all of which are the risk factors of dysphagia [15,35]. In patients affected by COVID-19, one must be aware of the existence of OD, even in the absence of intubation, and its association with increased morbidity and mortality. The need to incorporate swallow-function assessment as part of the daily clinical routine in older patients with COVID-19 affected with malnutrition and/or sarcopenia has been recommended [16]. On the other hand, the contribution of SARS-CoV-2 infection to the development of OD is presumed to be related to loss of taste and smell, common neurological symptoms in COVID-19 patients, as well as peripheral and central nerve invasion by the virus that may affect sensorimotor swallowing function [6,10,11,12], and is probably related to glossopharyngeal and vagal sensory neuropathy [6,36] and to the functional, nutritional, neurological and general health deterioration caused by the disease [6,10]. We have previously studied the role of pharyngeal sensory alterations in the pathophysiology of OD and found that there is an impaired cortical conduction and integration of pharyngeal sensory afferents in post-stroke and older patients with OD [37,38,39], concluding that pharyngeal sensory information is of key relevance in its pathophysiology. In our present study, prevalence and new cases of OD on admission in the three waves of the pandemic was very high. However, the prevalence was significantly higher in the first wave (51.7% *p* < 0.001 vs. second 31.3% and third wave 35.1%). On discharge, swallowing impairment was still present in 43.8%, 28.6% and 34.4 of patients from the first, second and third waves, respectively, and in our first longitudinal study, prevalence of OD remained high during the 6-month follow up (23.3%) [8], showing OD is a major issue among COVID-19 patients. A comparative study between the first two waves of the pandemics performed in the Sunnyview Rehabilitation Hospital in USA found a similar prevalence of OD on admission in the first wave (47.1% vs. 51.7% in our study) and it was significantly reduced in the second wave (13.8%), which included patients significantly younger [40]. As in our case, the reduced prevalence in the second and third waves could be related to patient’s age, as those in the second were younger compared to the first and third, to better management of patients, to the lower prevalence of neurological symptoms and use and need for high-flow oxygen and venturi masks which were more necessary in the first wave due to greater clinical severity, higher admission to the ICU and longer hospital stay. However, other studies have reported a lower prevalence of OD in a similar cohort of patients (28.9%) [9] and in post-extubated patients with COVID-19 (26.9%) [41]. These differences between our study and these others could also be related to the use of different methodologies to assess OD, or to different phenotypical characteristics of COVID-19 patients. The clinical test used (V-VST) in this study has excellent psychometrics (sensitivity 93.17%, specificity 81.39%, and inter-rater reliability Kappa = 0.77) [26,42] and was specifically adapted for the evaluation of COVID-19 patients [8].

In the first days of hospitalization, MN is common in COVID-19 due to appetite loss, systemic inflammation with increased hypermetabolism and muscle catabolism, and prolonged bed rest periods with disuse atrophy that is associated with muscle loss [33,43]. Our study identified many of these pathophysiological elements in the three waves of the pandemic, including anorexia (23.8%), vomiting and nausea (8.9%), diarrhea (30.1%) and reduced food intake (40.5%); respiratory failure requiring orotracheal intubation or ventilation with nasal intermittent positive pressure ventilation (12.2%); catabolic changes due the host inflammatory response phase; as well as high acute inflammation with CRP values between 7.7 and 10.2 mg/dL. Other authors have also reported 40% of patients with COVID-19 experiencing gastrointestinal symptoms such as nausea, vomiting, anorexia and diarrhea that can lead to MN [44], and that reduced food intake in these patients was associated with negative clinical outcomes [45]. Nutritional risk on admission in the patients in our series was extremely high (>98% in the 3 waves) according to the NRS-2002, a tool that has been validated to screen the risk of MN in hospitalized patients [30]. Other studies have found a similar percentage of patients with COVID-19 at nutritional risk (84.7–92%) [46,47], confirming the high impact of SARS-CoV-2 infection on nutritional status. On the other hand, assessment of MN with GLIM, recommended for COVID-19 patients [30], showed a high prevalence of MN in the first wave (45.9%) that was slightly reduced in the second (36.8%) and in the third (34.7; *p* < 0.05 vs. 1st wave). Regarding WL, we found highly significant differences when comparing the three waves: during the first wave, 36.8% of patients lost more than 6 kg during hospitalization with a mean WL of 6.5 ± 5.8 kg while these values were only 6.9% and 3.3 ± 4.2 kg, and 7.7% and 4.1 ± 4.3 kg in the second and third waves, respectively (percentage: *p* < 0.001; WL: 0.036). A review by Anker MS et al. 2021 [43] that included 589 patients from three studies reported a clinically notable WL (≥5%) in 37% of patients affected by COVID-19 during the first wave of the pandemic in 2020. Furthermore, we observed low albumin values and high levels of CRP and ferritin on admission among the three waves, as reported in other studies in 2021 [46]. Inflammatory status (CRP levels) improved on hospital discharge, as has been previously described [46,48]. These relevant improvements in nutritional status between the first, second and third waves, especially the impact on WL, are probably related to the implementation of a new nutritional systematic strategy “screen and treat” in COVID-19 wards after the first wave experience and that prioritized early nutritional supplementation before any assessment for MN. This new nutritional protocol, implemented from the second wave onwards, enabled an early, fast and generalized action to be taken. In the first wave we found that more than 95% of patients were at nutritional risk on admission, with 45% of patients diagnosed with malnutrition on discharge. The majority of patients were admitted with gastrointestinal symptoms, loss of appetite and previous WL, which required a quick response. For this reason, the “COVID-19 diet” was modified to be hypercaloric and hyperproteic and, in addition, all patients received two ONS a day systematically. They were then assessed during the first 24–48 h, and the diet was modified if necessary. This new protocol enabled generalized intervention in all patients admitted to our hospital with COVID-19. This kind of early and intensive nutritional treatment for COVID-19 patients has been recently recommended in a review on the topic, stating that “(t)argeted nutritional therapy should be started early in severe illness and sustained through to recovery if clinical and patient-centered outcomes are to be optimized” [33]. In addition, we have previously developed and applied a similar early intervention based on compensatory treatment for acute older patients with OD in order to reduce nutritional and respiratory complications. It is defined as the Minimal Massive Intervention (MMI), and is based on our previous scientific evidence and consists of: (a) fluid thickening (250 mPa·s and 800 mPa·s) [27] and textured modified diets (fork mashable or pureed diet) [32]; (b) caloric and protein ONS; and (c) oral health and hygiene recommendations during hospitalization and on discharge. Since the implementation of the MMI, there has been an improvement in nutritional status and functionality and a reduction in hospital readmissions, respiratory infections and mortality (NCT04581486) [8].

The clinical spectrum of patients infected with SARS-CoV-2 varies from mild clinical involvement to severe hypoxemia and pulmonary infiltrates. Up to 62% of patients in the study sample developed interstitial pneumonia, with significant differences between the second and third waves (68.0% vs. 57.2%; *p* < 0.05). Other studies have also reported high incidence of interstitial pneumonia, ranging from 53% to 91% [3,49]. These cases can worsen prognosis by pulmonary overinfection, due to bacteria residing in the oral cavity, which are capable of producing an additional bacterial pneumonia, the main etiological mechanism of which is aspiration, common in patients with OD. In our study, we found a prevalence of bacterial overinfection of 12.4%, 13% and 6.7% in the first, second, and third waves, respectively (*p* = 0.50). Regarding ICU admission, 8.2% of patients were admitted to ICU (*p* < 0.05 first wave vs. second and third), with a mean stay of 15.5 days (*p* < 0.001 first wave vs. second and third). We cannot attribute these higher figures during the first wave to the severity of the disease alone but also to the initial lack of knowledge in the management of COVID-19 by clinical staff. Other studies showed a higher percentage of ICU admission (17% and 26%) [3,49,50] with a similar median stay of 14 days. Of those patients in our study admitted to the ICU, 6.5% needed orotracheal intubation with the highest rate also in the 1st wave (10.7% vs. 5.1% 2nd wave, *p* < 0.05; and 3.5% 3rd wave, *p* < 0.01). Regarding hospital discharge, our patients remained at the hospital for a mean of 12.7 days, slightly less than that reported in a systematic review, which showed a mean of 14 (10–19) days [51]. Mortality found in the third wave was higher than in the first one (*p* < 0.01 vs. 1st wave); however, in the first wave we were not able to assess all patients in the first 24–48 h and many of them were not included in the study because they were admitted to the ICU very early and thus mortality in this wave is probably underestimated. In contrast, during the second and third waves, the screening and assessment was more systematic and exhaustive, and we ensured that almost all patients had both screening and assessment including those who were admitted to the ICU or died early, therefore increasing the mortality rates of our study. Other studies have shown disparate mortality prevalences: 34.0% during the first wave in Canada [49]; China (11.0%) [3]; and Spain (11.4%, 19.8% and 18.0%) [52,53,54]. Our mortality results are in line with these ranges.

Finally, we found that older age and poorer functional status were relevant indicators among COVID-19 patients and independently associated with OD and MN. In addition, each one of these conditions (swallowing impairment and poor nutritional status) was independently associated with each other, indicating their close relationship and the relevance of an early screening and treatment for both in order to improve patient general health status and avoid secondary complications. These results are in accordance with our previous studies on patients without COVID-19 with OD [8,55,56,57] as well as with other authors [58,59,60]. Regarding intrahospital mortality, we found that old age, OD and MN were independently associated with this outcome indicating that a vulnerable status probably related to frailty could facilitate the non-recovery of the acute disease leading to this fatal outcome. A plausible reason for this is that these patients present age-related chronic medical conditions, frailty, comorbidities or sarcopenia [61] and/or lower immunity levels [61]. In addition, the age-dependent defects in B-cell and T-cell function and the excess production of type-2 cytokines could lead to prolonged proinflammatory responses and deficiency in control of viral replication, potentially leading to poor outcome [62]. OD and MN also play a key role in the functional and health deterioration of individuals as they are not able to safely acquire nutrients nor liquids to achieve the nutritional and hydric needs of the organisms, especially in a severe acute situation such as COVID-19 disease [33].

## 5. Conclusions

The COVID-19 patient phenotype in our sample was an adult patient over sixty years of age who came from the community and presented slight functional impairment before infection. On hospital admission, the prevalence of interstitial pneumonia was very high, as was the need for intensive respiratory therapy. Prevalence of OD, MN, severe loss in body weight, and mortality was also very high. In our study, OD was independently associated with MN and mortality. An early, systematic and proactive multimodal nutritional intervention including fluid thickening, texture modified diets and nutritional support improved patients’ nutritional status. OD and MN are major issues among hospitalized COVID-19 patients and management of these two conditions should be prioritized and included in therapeutic protocols and guidelines for COVID-19 patients in order to improve their clinical outcomes during hospitalization.

## Figures and Tables

**Table 1:** Demographic characteristics of the first three waves of COVID-19 patients.

	1st Wave	2nd Wave	3rd Wave	*p*-Value
**Mean age (years)**, ±SD	69.3 ± 17.6	66.4 ± 17.5 ^ŦŦ^	72.0 ± 16.3	**0.004**
**Sex (% female)**, n/N	52.2 (107/205)	45.5 (91/200)	52.0 (104/200)	0.311
**Patient residence**, %				
Community (n/N)	66.8 (137/205)	90.5 (181/200) ***	83.5 (167/200) ***	**<0.001**
Nursing home (n/N)	29.8 (61/205)	9.0 (18/200)	15.0 (30/200)	
Intermediate health center (n/N)	3.4 (7/205)	0.5 (1/200)	1.5 (3/200)	
**Mean Barthel Index (admission)**, ±SD	73.3 ± 33.6	80.2 ± 31.8	74.0 ± 33.9	**0.012**
Independent (100%) (n/N)	51.1 (92/180)	63.0 (126/200) *	53.6 (105/196)	0.112
Moderate dep. (61–95%) (n/N)	15.0 (27/180)	14.5 (26/200)	13.3 (26/196)	
Severe Dep. (21–60%) (n/N)	22.8 (41/180)	12.0 (24/200)	20.9 (41/196)	
Total Dep. (<20%) (n/N)	11.1 (20/180)	10.5 (21/200)	12.2 (24/196)	
**Main neurological symptoms**, %				
Confusion (n/N)	40.0 (82/205)	14.6 (29/199) *** ^ŦŦŦ^	28.5 (57/200) *	**<0.001**
Headache (n/N)	28.3 (58/205)	24.6 (49/199)	17.5 (35/200) *	**0.034**
Delirium (n/N)	15.1 (31/205)	3.5 (7/199) ***	7.5 (15/200) *	**<0.001**
Ageusia (n/N)	4.4 (9/205)	3.0 (6/199)	2.0 (4(200) *	0.384
Anosmia (n/N)	3.4 (7/205)	2.5 (5/199)	0.5 (1/200)	0.118
Encephalitis (n/N)	2.0 (4/205)	0.5 (1/199)	0 (0/200) *	**0.079**

* *p* < 0.05 vs. 1st wave; *** *p* < 0.001 vs. 1st wave; ^ŦŦ^ *p* < 0.01 vs. 3rd wave; ^ŦŦŦ^ *p* < 0.001 vs. 3rd wave. Bold indicates statistically significant. Dep. indicates dependence.

**Table 2:** Clinical characteristics and therapeutic approach of the three waves of COVID-19 patients.

	1st Wave	2nd Wave	3rd Wave	*p*-Value
**Interstitial pneumonia**, % (n/N)	61.0 (125/205)	68.0 (131/193) ^Ŧ^	57.2 (111/194)	0.09
**Nasal goggle**, % (n/N)	73.7 (151/205)	84.3 (167/198) **	85.8 (169/197) **	**0.003**
**Facial mask**, % (n/N)	42.0 (86/205)	43.9 (87/198)	40.6 (80/197)	0.797
**High concentration oxygen mask**, % (n/N)	26.3 (54/205)	22.7 (45/198)	23.2 (46/198)	0.66
**Venturi mask (high flux)**, % (n/N)	37.7 (77/204) ^ŦŦŦ^	29.3 (58/198) ^ŦŦ^	17.2 (34/198)	**<0.001**
**NIPPV**, % (n/N)	3.1 (6/196)	7.6 (15/197) *	7.1 (14/198)	0.113
**OTI**, % (n/N)	10.7 (22/205)	5.1 (10/197) *	3.5 (7/198) **	**0.008**
**OTI Days** (mean ± SD)	13.4 ± 9.7	8.8 ± 4.8	24.4 ± 25.9	0.062
**FiO2 max** (mean ± SD)	41.4 ± 33.2	51.8 ± 29.6 ***	50.9 ± 30.3 ***	**0.043**
**PAFI** (mean ± SD)	248.1 ± 140.6	225.8 ± 114.2	235.1 ± 110.5	**0.004**
**ICU Admission**, % (n/N)	12.2 (25/205)	6.1 (12/198) *	6.1 (12/198) *	**0.034**
**ICU stay days** (mean ± SD)	15.0 ± 9.8	11.3 ± 4.3	20.8 ± 23.7	0.544
**Hospital stay Days** (mean ± SD)	16.8 ± 13.0	11.2 ± 7.7 *** ^Ŧ^	10.5 ± 10.7 ***	**<0.001**
**Mortality**, % (n/N)	10.7 (22/205)	13.6 (27/199)	19.1 (38/199) *	0.052

NIPPV: nasal intermittent positive pressure ventilation; OTI: orotracheal intubation; FiO2: fraction of inspired oxygen; PAFI (PaO2/FiO2): ratio between oxygen arterial pressure (PaO2) and the inspired fraction of oxygen (FiO2); ICU: intensive care unit. * *p* < 0.05 vs. 1st wave; ** *p* < 0.01 vs. first wave; *** *p* < 0.001 vs. first wave; ^Ŧ^ *p* < 0.05 vs. third wave; ^ŦŦ^ *p* < 0.01 vs. third wave ^ŦŦŦ^ *p* < 0.001 vs. third wave. Bold indicates statistically significant. Underlined means nearly reaches statistical significance.

**Table 3:** Swallowing assessment of the three waves of COVID-19 patients.

	1st Wave		2nd Wave		3rd Wave		*p*-Value	
**OD SCREENING**								
**EAT-10** (mean ± SD)	0.8 ± 1.6		0.5 ± 1.2 ^Ŧ^		0.2 ± 0.9 ***		**<0.001**	
**Previous diagnosis of OD**, % (n/N)	9.8 (20/205)		13 (26/200)		9.0 (18/200)		0.384	
**SPECIFIC ANAMNESIS OF OD**								
**Difficulty eating or drinking?** (% yes) (n/N)	48.3 (99/205)		31.6 (61/193) ***		30.2 (54/179) ***		**<0.001**	
**Do you cough or choke when you drink liquids?** (% yes) (n/N)	43.4 (89/205)		23.3 (45/193) ***		21.2 (38/179) ***		**<0.001**	
**Do you cough or choke when you drink thick liquids?** (% yes) (n/N)	0.5 (1/203)		1.0 (2/193)		1.1 (2/179)		0.769	
**Do you have difficulty chewing or swallowing solids?** (% yes) (n/N)	50.2 (103/205)		35.2 (68/193) **		35.8 (64/179) **		**0.003**	
**Do you have difficulty chewing or swallowing an omelette?** (% yes) (n/N)	11.2 (23/205)		13.5 (26/193)		15.6 (28/179)		0.445	
**OD CLINICAL ASSESSMENT (V-VST)**								
	**ADM**	**DISCH**	**ADM**	**DISCH**	**ADM**	**DISCH**	**ADM**	**DISCH**
**OD**, % (n/N)	51.7 (106/205)	43.8 (88/201)	31.3 (62/198) ***	28.6 (56/196) **	35.1 (66/188) ***	34.4 (66/192)	**<0.001**	**0.006**
Impaired efficacy, % (n/N)	48.0 (98/204)		29.8 (59/198) ***		37.2 (70/188) *		**<0.001**	
Impaired safety, % (n/N)	44.1 (90/204)		22.2 (44/198) ***		27.1 (51/188) ***		**<0.001**	
**FLUID ADAPTATION AND TEXTURE MODIFIED DIETS ON ADMISSION**								
**FLUID ADAPTATION**								
<50 mPa·s	55.6 (114/205)		81.0 (162/200) ***		74.9 (149/199) ***		**<0.001**	
250 mPa·s	39.0 (80/205)		15.0 (30/200)		21.1 (42/199)			
800 mPa·s	4.9 (10/205)		3.0 (3/200)		3.5 (7/199)			
**TEXTURE MODIFIED DIETS**								
Normal diet	45.9 (94/205)		59.0 (118/200) *		49.5 (98/198)		0.074	
Fork mashable	26.3 (54/205)		21.5 (43/200)		22.2 (44/198)			
Puree	27.3 (56/205)		18.5 (37/200)		28.3 (56/198)			
**FLUID ADAPTATION AND TEXTURE MODIFIED DIETS ON DISCHARGE**								
**FLUID ADAPTATION**								
<50 mPa·s	78.1 (143/183)		88.2 (150/170) **		85.7 (138/161)		**0.012**	
250 mPa·s	16.4 (30/183)		8.2 (14/170)		12.4 (20/161)			
800 mPa·s	4.9 (9/183)		1.2 (2/170)		0.6 (1/161)			
**TEXTURE MODIFIED DIETS**								
Normal diet	60.7 (111/183)		67.6 (115/170)		67.7 (109/161)		0.320	
Fork mashable	23.5 (43/183)		21.2 (36/170)		18.6 (30/161)			
Puree	15.3 (28/183)		8.8 (15/170)		12.4 (20/161)			

OD: oropharyngeal dysphagia; EAT-10: Eating Assessment Tool-10; V-VST: volume-viscosity swallowing test; ADM: admission; DISCH: discharge. * *p* < 0.05 vs. first wave; ** *p* < 0.01 vs. first wave; *** *p* < 0.001 vs. first wave; ^Ŧ^ *p* < 0.05 vs. third wave. Bold indicates statistically significant. Underlined means nearly reaches statistical significance.

**Table 4:** Nutritional screening and assessment of the three waves of COVID-19 patients.

	1st Wave	2nd Wave	3rd Wave	*p*-Value
**MN SCREENING**				
**Risk of MN (NRS2002** **≥ 3)**, ±SD	3.1 ± 1.0	3.3 ± 1.2	3.2 ± 0.9	0.238
No risk of MN, % (n/N)	0.5 (1/203)	1.6 (3/189)	1.3 (2/153)	0.560
At risk of MN, % (n/N)	99.5 (202/203)	98.4 (186/189)	98.7 (151/153)	
**NUTRITIONAL ASSESSMENT**				
**MN GLIM Criteria**, % (n/N)	45.9 (89/194)	36.8 (71/193)	34.7 (67/193) *	0.057
**Mean BMI (kg/m^2^)** ± SD	28.5 ± 5.4	29.2 ± 6.4	28.3 ± 5.0	0.510
**Main symptoms (%) (on admission)**				
Anorexia, % (n/N)	29.3 (39/133)	21.7 (36/166)	34.7 (67/193) *	0.207
Vomiting/nausea, % (n/N)	16.6 (34/205)	7.1 (14/198) ** ^Ŧ^	2.6 (5/195) ***	**<0.001**
Diarrhea, % (n/N)	30.1 (73/205)	20.2 (40/198) *** ^ŦŦŦ^	7.2 (14/195) ***	**<0.001**
Incomplete diet intake, % (n/N)	41.9 (57/136)	39.2 (65/166)	40.8 (62/152)	0.886
**ONS prescription**, (% yes) (n/N)	52.2 (95/182)	79.3 (153/193) *** ^ŦŦ^	89.5 (170/190) ***	**<0.001**
**NGT placement**, (% yes) (n/N)	16.9 (143/172)	7.7 (15/194) *	4.2 (8/189) ***	**<0.001**

MN: malnutrition; GLIM: global leadership initiative on malnutrition; BMI: body mass index; ONS: oral nutritional supplements; NGT: nasogastric tube. * *p* < 0.05 vs. first wave; ** *p* < 0.01 vs. first wave; *** *p* < 0.001 vs. first wave; ^Ŧ^ *p* < 0.05 vs. third wave; ^ŦŦ^ *p* < 0.01 vs. third wave; ^ŦŦŦ^ *p* < 0.001 vs. third wave. Bold indicates statistically significant. Underlined means nearly reaches statistical significance.

**Table 5:** Weight loss comparative between the three waves of COVID-19 patients.

	1st Wave	2nd Wave	3rd Wave	*p*-Value
**Weight loss (%) (on admission)**				
>10 kg	19.2 (24/125)	1.5 (2/130) ***	1.7 (2/116) ***	**<0.001**
6–10 kg	17.6 (22/125)	5.4 (7/130)	6.0 (7/116)	
3–6 kg	20.8 (26/125)	10.0 (13/130)	11.2 (13/116)	
1–3 kg	22.4 (28/125)	33.1 (43/130)	37.1 (43/116)	
None	20.0 (25/125)	50.0 (65/130)	44.0 (51/116)	
**Weight loss (kg)** (mean ± SD)				
Pre-admission to admission	2.3 ± 3.2	2.1 ± 2.7	1.8 ± 2.4	**0.004**
Admission to discharge	4.4 ± 5.4	0.9 ± 3.2 ***	1.5 ± 3.1 ***	0.055
**TOTAL WEIGHT LOSS (kg)** (mean ± SD)	6.5 ± 5.8	3.3 ± 4.2 ***	4.1 ± 4.3 **	**0.036**

** *p* < 0.01 vs. first wave; *** *p* < 0.001 vs. first wave. Bold indicates statistically significant. Underlined means nearly reaches statistical significance.

**Table 6:** Weight loss comparative between the three waves of COVID-19 patients.

	1st Wave		2nd Wave		3rd Wave		*p*-Value	
	**ADM**	**DISCH**	**ADM**	**DISCH**	**ADM**	**DISCH**	**ADM**	**DISCH**
**Albumin** (g/dL)	3.3 ± 0.5	3.3 ± 0.7	3.4 ± 0.4	3.2 ± 0.5	3.4 ± 0.4	3.1 ± 0.7	0.54	0.409
**Total proteins** (g/dL)	6.5 ± 0.7	6.1 ± 0.8	6.7 ± 0.6 ^ŦŦŦ^	6.2 ± 0.9	6.4 ± 0.7	6.1 ± 0.6	**<0.001**	0.506
**Lymphocytes** (1 × 10^3^ µL)	1.3 ± 0.7 ^ŦŦŦ^	1.8 ± 1.6	1.2 ± 0.6 ^Ŧ^	1.5 ± 0.8 **	1.1 ± 1.1	1.7 ± 1.9	**<0.001**	**0.008**
**Cholesterol** (mg/dL)	136.7 ± 42.7	186.7 ± 58.5	149.1 ± 39.6	149.5 ± 41.1 *	124.8 ± 30.1	156.2 ± 47.4 *	0.161	**0.011**
**Ferritin** (ng/mL)	987.7 ± 1331.2	889.0 ± 1188.0	785.2 ± 854.7	1271.2 ± 4894.9	815.6 ± 887.4	873.3 ± 1148.3	0.389	0.729
***C*-reactive protein** (mg/dL)	10.2 ± 9.9	2.8 ± 4.9 ^Ŧ^	7.7 ± 6.2	3.1 ± 5.4 ^Ŧ^	7.7 ± 7.3	3.9 ± 6.2	0.155	**0.010**

ADM: admission; DISCH: discharge. * *p* < 0.05 vs. first wave; ** *p* < 0.01 vs. first wave; ^Ŧ^ *p* < 0.05 vs. third wave; ^ŦŦŦ^ *p* < 0.001 vs. third wave. Bold indicates statistically significant.

**Table 7:** Multivariate logistic regression analysis of risk factors associated with oropharyngeal dysphagia on discharge, malnutrition and mortality.

	*p*-Value	OR (95% CI)
**Oropharyngeal dysphagia on discharge**		
Age	**<0.0001**	5.16 (2.44–10.90)
Delirium	**<0.0001**	7.09 (2.84–17.69)
Risk of malnutrition (NRS-2002 ≥ 3)	**<0.0001**	5.16 (2.44–10.90)
Malnutrition	**0.001**	2.13 (1.39–3.26)
**Malnutrition**		
Age	**<0.0001**	0.17 (0.12–0.26)
Incomplete diet on discharge	**<0.0001**	4.94 (2.99–8.16)
Oropharyngeal dysphagia	**<0.0001**	3.16 (1.83–5.48)
Diarrhea	**0.001**	2.74 (1.52–4.92)
ICU admission	**0.001**	4.79 (1.91–11.98)
**Mortality**		
Age	**<0.0001**	0.01 (0.00–0.47)
Malnutrition	**<0.0001**	5.40 (2.69–10.82)
Oropharyngeal dysphagia	**<0.0001**	4.40 (2.23–8.69)

ICU: Intensive care unit. Bold indicates statistically significant.

## Data Availability

Not applicable.

## Supplementary Materials

The following supporting information can be downloaded at: https://www.mdpi.com/article/10.3390/nu14183826/s1, File S1: Post-COVID-19 recovery diet.
