# Supplementary material for: A Comparative Study between the Three Waves of the Pandemic on the Prevalence of Oropharyngeal Dysphagia and Malnutrition among Hospitalized Patients with COVID-19
Source: Nutrients. 2022 Sep 16;14(18):3826. doi: 10.3390/nu14183826 (PMC9502091; doi:10.3390/nu14183826)
Supplement: Supplementary file 1 [file nutrients-14-03826-s001.zip › Supplementary file S1.pdf]

Post-COVID-19 recovery diet

GENERAL RECOMMENDATIONS

- Temporarily, and until they recover their usual mobility (muscle mass and strength), it is necessary to follow a diet richer in protein (dairy products, meat, fish, eggs, pulses).
- It is also important to maintain or start rehabilitation exercises to regain muscle mass.
- It would not be optimal to lose more weight. It is therefore advisable to weigh yourself every week to monitor your progress. In case of weight loss, contact the dietician-nutritionists (phone number - extension XXXX).

DIETARY RECOMMENDATIONS

MEAL	FOODS
**BREAKFAST**	**Dairy product / soya drink +** bread **+ tuna / omelette / boiled ham**
**MID-MORNING**	**1 high-protein yoghurt*** + 1 fruit
**LUNCH**	Pasta / rice / pulses (3-4 times a week) / potato<br>**Meat / fish / eggs**<br>Vegetables / salad<br>Fruit
**SNACK**	**1 high-protein yoghurt* or cottage cheese** + 1 fruit
**DINNER**	Vegetables / salad<br>Pasta / rice / potato<br>**Meat / fish / eggs**<br>Fruit
**SNACK**	**1 glass of milk/soy drink**<br>If protein intake is low, add 2 tablespoons of skimmed milk powder**.

Recommended proportion at lunch and dinner:

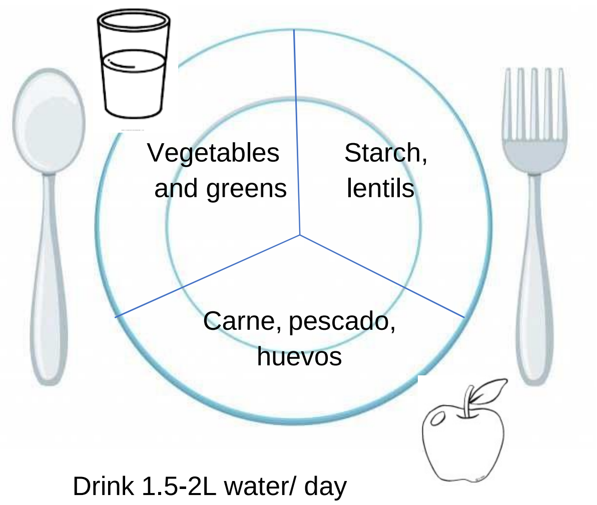

Drink 1.5-2L water/ day

*High-protein yoghurt sold in supermarkets: yoghurts with a protein content of more than 10g/100g of food, e.g. Skyr (LIDL), Yo-Pro (Danone), + Proteins (Mercadona)...

**Skimmed milk powder sold in supermarkets.

Eliminate or limit the following foods to occasional consumption and moderate amounts:

- ***SAUSAGES and SWEETS:*** *smoked sausage, salami... and pastries, industrial and homemade pastries...*
- ***SUGARED DRINKS and ALCOHOLIC BEVERAGES:*** *soft drinks, fruit juices, wine, beer...*

In order to ensure protein intake and use, in addition to the recommended protein intake, it is possible to:

Immediately after the rehabilitation exercises, take an extra protein intake: a high-protein yoghurt, milk enriched with milk powder, cottage cheese, tuna sandwich.

If you find it difficult to chew and/or swallow meat and fish, you can eat it in minced form or in soft preparations such as cannelloni, meat pies, puddings, omelettes...
